# Mining of risk perception dimensions of Chinese tourists’ outbound tourism based on word vector method

**DOI:** 10.3389/fpsyg.2022.1091065

**Published:** 2022-12-22

**Authors:** Caihua Yu, Yilin Zhao

**Affiliations:** School of Management Science and Engineering, Nanjing University of Information Science and Technology, Nanjing, China

**Keywords:** mining, risk perception dimensions, outbound tourism, China, word vector method

## Abstract

**Introduction:**

Safety is the lifeline of tourism development. The article’s goal is to examin how Chinese tourists perceive risk when travelling aboard.

**Methods:**

In order to create the initial corpus, this study first uses “outbound tourism“as the key word to crawl the question and answer (hereinafter referred to as “Q & A”) data from 4 Chinese online travel operator platforms, then preprocesses the “Q & A” data in Python. Secondly, after being extracted, the feature words are converted into the word vector model using the word vector method based on neural network language model. Finally, the word vectors are clustered and classified.

**Results:**

It is found that there are six dimensions of risk perception of Chinese tourists’ outbound tourism, namely traffic risk, planning risk, service risk, communication risk, financial risk and functional risk.

**Discussion:**

Important and practical information for government and tourism enterprises is provided to accurately grasp the risk perception of Chinese tourists’ outbound tourism and continuously improve the supply of tourism risk information.

## 1. Introduction

As Chinese residents’ living standards are improved and the concept of leisure consumption is upgraded, the scale of outbound tourism is expanding. In 2012, the number of Chinese outbound tourists ranked first in the world for the first time, then increased steadily (China Tourism Academy, 2020). At the same time, due to the characteristics of outbound tourism activities such as remoteness, strong mobility, language barriers, etc., the influencing factors of tourism safety are complex and diverse, which escalates the frequency of outbound tourism risks and increases the degree of harm (Wu, 2017). For example, in 2019, in the lobby of the budget hotel Kyriad in Vitry-sur-Seine in the 94th province of Paris, a female Chinese tourist who had just arrived in France with a tour group was robbed by two masked robbers (Huang, 2019). Property damage is estimated to cost 40,000 euros. Aside from objective reasons, a lack of safety awareness and self-protection awareness are important factors that contribute to frequent outbound travel safety incidents among Chinese tourists. To ensure the safety of Chinese tourists’ outbound travel, the Chinese government and enterprises must urgently clarify the current situation of Chinese tourists’ outbound tourism risk perception, provide targeted tourism risk warning information services for Chinese tourists, and guide them to improve their safety awareness and self-protection.

Tourism risk perception refers to tourists’ intuitive understanding of the probability that actual risks may occur during the travel process, which directly affects tourists’ travel decisions and behaviors (Loewenstein et al., 2001). Existing related studies often preset the dimensions of risk perception, by using data from questionnaire surveys, and then use statistical analysis methods such as principal component analysis or the factor analysis to explore the dimensions of risk perception (Rufat and Botzen, 2022; Xue et al., 2022). The above methods cannot remove the subjective judgment of researchers, so these scholars cannot accurately start from the psychological feelings of tourists, that is, they cannot dig out the objective dimension of tourism risk perception. Furthermore, these methodologies have trouble dealing with suitably big sample sizes, which may jeopardize the study’s results. With the rapid development of the Internet and social media, a large amount of information about the risks of Chinese tourists traveling abroad has flooded the Internet. For example, the Ministry of Culture and Tourism of the People’s Republic of China and the Ministry of Foreign Affairs of the People’s Republic of China have all provided outbound travel risk warning information on their official websites. Online travel operator platforms such as Ctrip.com and Tuniu.com have gathered online commentary information from many tourists on the risks of outbound travel. In particular, the “Q & A” section (a place that specializes in providing consulting services for tourists) provided by some operators gathers many tourists’ questions and answers about outbound travel. These “Q & A” data are the most real risk perception before and after the tourists’ outbound travel. In addition to survey data, they are another important data source for grasping the dimensions of Chinese tourists’ outbound tourism perception risk.

Online reviews of tourists have the typical characteristics of big data, namely “large volume, various modalities, velocity, and great value but low density” (referred to as “4 V”) (Mayer-Schönberger and Cukier, 2013). With the rapid development of neural networks and deep learning, word vectors have become a hot topic in natural language processing in order to overcome the challenges of the “4 V” features of big data. Neural network was applied in language modeling (Bengio et al., 2003). Mikolov et al. (2013) proposed the word vector concept Word2Vec, the core idea of which is to learn the vector expression of words through context (Bengio et al., 2003). Word2vec has had a huge impact on deep learning (Church, 2017), and is widely used in product recommendations and the sentiment analysis and automatically extraction of semantic topics. Baek and Chung (2021) proposed the multimedia recommendation method using Word2Vec-based social relationship mining. Esmeli et al. (2020) investigated the applicability of “word2vec and clustering based text representation” method for Twitter sentiment analysis. Haider et al. (2020) proposed a sentence based clustering algorithm (K-Means) for a single document, and they have used Gensim word2vec which is intended to automatically extract semantic topics from documents in the most efficient way possible.

With the “Q & A” data of tourists on online travel operator platforms such as Ctrip.com and Tuniu.com, this paper mines the outbound tourists’ travel risk perception dimensions with the world vector method. Compared with previous studies, the theoretical and practical contributions of this study are as follows: First, it examines the dimensions of outbound travel risk perception for Chinese tourists utilizing fresh data, especially “Q & A” data gathered from travelers on websites run by online tour operators like Ctrip.com and Tuniu.com. Second, by employing the word vector approach to mine the dimensions of tourist risk perception, it advances previously used statistical analytical techniques like principal component analysis and the factor analysis. Third, in terms of information discovery, it offers data support for the division of Chinese visitors’ outbound tourism risk perception aspects. Fourth, it helps the government and tourism businesses precisely understand how tourists perceive danger in different contexts, and it keeps the flow of information on risk improving.

## 2. Literature review

Risk perception refers to the individual’s psychological feelings of objective risks existing in the outside world, and emphasizes the influence of individual experience obtained from intuitive judgment and subjective feelings on cognition (Bauer, 1960). Tourism risk perception is the research of risk perception in the tourism context. However, due to the subjectivity of tourists’ perception and the uncertainty of risk, tourism risk perception is difficult to be accurately defined and measured.

Tourism risk perception is the probability of risk occurrence (Loewenstein et al., 2001). It believes that tourism risk perception refers to tourists’ intuitive understanding of the probability of actual risks in the tourism process (Lepp and Gibson, 2003). Tourism risk perception is the sum of the severity and probability of the results (Le and Arcodia, 2018). Tourism risk perception can be interpreted as tourists’ subjective judgment of tourism risk, and the core content is the harm of uncertainty and consequences (Moutinho et al., 2011). It originates from product internal, purchase location and mode, economic and social psychology, and tourists own experience. Uncertainty and harmful consequences are two common methods to measure tourism risk perception. Perceived severity and vulnerability are used to study the attitude of risk perception (Wang et al., 2019).

Tourism risk perception includes some specific contents such as financial risk and physical risk, which is called tourism risk perception dimensions and also known as types or dimensions (Le and Arcodia, 2018). Tourism risk perception dimension is not limited to certain aspects (Zhang, 2009; Li et al., 2014; Hasan et al., 2017). Tourism risk perception was initially divided into equipment risk, financial risk, physical risk, psychological risk, satisfaction risk, social risk and time risk (Roehl and Fesenmaier, 1992), which was also the starting point for many scholars to explore the dimensions of tourism risk perception. On this basis, the perceived dimensions of outbound tourism risk include health risk, traffic risk (Hussain, 2023), political risk (Chi, 2020), terrorism risk, dietary risk, cultural barrier risk, religious risk and criminal risk (Lepp and Gibson, 2003; Li et al., 2015). Later, some scholars increased the expected risk and psycho-social risk (Adam, 2015; Kong and Zhu, 2021).

The mining methods of tourism risk perception dimension are limited, mainly as follows: (1) Meta-analysis of tourism risk perception dimensions. According to the statistical analysis of the types and quantities of exit prompt risks published by the website of the China National Tourism Administration, tourists’ tourism risk perception dimensions can be divided into meteorological disasters, traffic accidents, animal invasions, criminal events, sudden diseases, geological disasters, cultural conflicts and food poisoning, and then takes Shanghai city as an example to measure urban residents’ exit tourism risk perception by questionnaire survey (Wu, 2017). (2) Exploring the tourism risk perception dimensions of the subjects through structural interview and the text analysis (Kong and Zhu, 2021). A cross-sectional design was used to examine the travel health risk perceptions of US study abroad students, and found that the top-rated threats being contaminated food/water, psychological distress, personal assault, and excessive sun exposure (Hartjes et al., 2009). (3) Principal component analysis and exploratory factor analysis. For example, the questionnaire was used to get the tourism risk perception of outbound tourists, and the factor analysis method was used to divide the tourism risk perception dimension of outbound tourists into terrorist events and war risks, public health, natural disasters and financial risks (Chen et al., 2009). Based on the literature analysis and the questionnaire survey, the exploratory factor analysis was used to find the domestic consumer tourism perceived risk dimension in addition to physical risk, functional risk, time risk, financial risk, social risk and psychological risk, also includes service risk, facility risk and communication risk (Xu et al., 2013). (4) Researchers presuppose dimensions of risk perception and make a questionnaire to verify the hypothesis. While studying the impact of high-altitude defense events on the tourism risk perception of Chinese residents traveling to South Korea, Zhang’s team measured the tourism risk perception from social stability, political security, conflicts and accidents, and insecurity (Zhang et al., 2020).

The researches of tourism risk perception dimensions present a trend of multi-perspective and diversification. However, because tourism risks are endogenous (Wu, 2017), dimensions of tourists’ perception risk varies with tourism locations, tourism time, tourism projects and other situations, so it is challenging to identify the dynamic tourism risk perception. Previous research on tourism risk perception of Chinese tourists often predetermined risk dimensions by analyzing literature, or acquired tourists’ subjective perception of a certain risk through the survey method. The above methods can simplify the procedure of dimension, but may lead to a problem, that is, the preset perceived risk dimensions are out of reality, and are not in line with the characteristics of the destination. Furthermore, these methodologies have trouble dealing with suitably big sample sizes, which may jeopardize the study’s results. Based on this, the research of risk perception of Chinese tourists’ outbound travel makes the following two improvements. (1) A reliable, unbiased data source. More accurately reflecting the degree of risk perception among visitors than the situational hypothesis data from the questionnaire, the online “Q & A” data is more dependable. (2) A context-based strategy. The word vector approach we utilize can examine the level of perception of danger from the psychological level more precisely than more conventional methods like principal component analysis and the factor analysis. Therefore, this article employs the word vector approach to analyze online “Q & A” data in order to examine the dimensions of Chinese visitors’ perceptions of outbound tourism danger. We hope to provide pioneering suggestions for the safe management of outbound tourism.

## 3. Materials and methods

### 3.1. Word vector method

Language model is an important part of natural language processing (NLP), and the word vector depends on the training of language model. Word vector method involves two important models: CBOW model and skip-grams model. CBOW model predicts wt under the premise of known w_t-2_, w_t-1_, w_t + 1_, w_t + 2_, i.e., the frequency of occurrence of the current word is predicted by context. Skip-grams model is just the opposite of CBOW model, which is to predict w_t-2_, w_t-1_, w_t + 1_, w_t + 2_ on the premise of known w_t_ (Ning and Liu, 2016).

**Figure 1:**
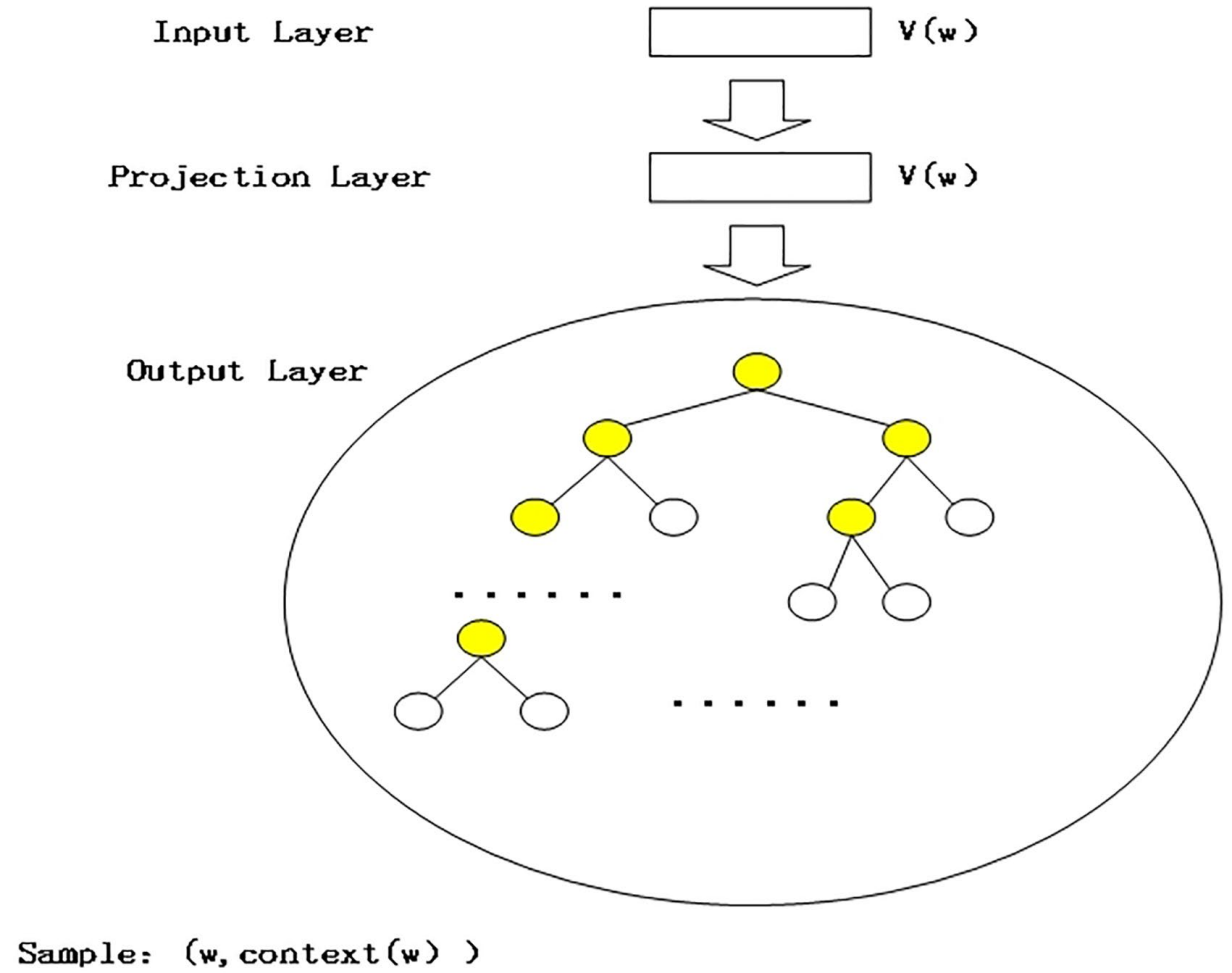
Skip–grams model based on Hierarchical Softmax.

This study uses Word vector method to find tourism risk perception dimensions. The word vector is constructed by using Python Gensim toolkit and the skip-grams model based on Hierarchical Softmax, as shown in Figure 1. The input layer contains only one word w, and the word vector v (w) ∈Rm; it projects v(w) onto v(w) by means of the projection layer. The projection is identical. In skip-grams model, the projection layer is actually redundant. The output layer is a Huffman tree. The word vector of the root node of Huffman tree is the word vector that is mapped by the projection layer. All the corresponding leaf nodes correspond to each word in the training corpus and the number of leaf nodes represents the number of words in the training corpus.

### 3.2. Framework and process

#### 3.2.1. Source of corpus, data crawling, and preprocessing

In December 2019, after searching the major online travel operator platforms one by one, it can be found that 8 platforms, including Ctrip.com, Mafengwo.com, Tuniu.com, and Qyer.com, have “Q & A” section, as detailed in Table 1.

**Table 1:** List of online travel operators with the “Q & A” section.

Name of Online Travel Operator	“Q & A” section (yes / no)	Name of Online Travel Operator	“Q & A” section(yes / no)	Name of Online Travel Operator	“Q & A” section(yes / no)	Number of “Q & A”
Qunar.com	No	Uzai.com	No	Ctrip.com	Yes	538,236
Wantu.cn	No	Tripadvisor.cn	No	Tuniu.com	Yes	1,073,111
Fliggy.com	No	Joytraveller.com	No	Lvmama.com	Yes	7,649
www.ly.com	No	Lotour.com	No	Mafengwo.com	Yes	4,626,016
Elong.com	No	Qmango.com	No	Qulxw.cn	Yes	2,759
Booking.com	No	Chinese National Geography	No	Qyer.com	Yes	0
Agoda.cn	No	UULUX Travel	No	Shijiebang.com	Yes	0
Njaihua.com	No	www.myantu.com	No	Youxiake.com	Yes	0
Mayi.com	No	www.yododo.cn	No	Toursforfun.com	No	
Ch.com	No	Zoutu.com	No	Miutour.com	No	
Xiaozhu.com	No	Zhuna.cn	No	Nilai.com	No	
Tujia.com	No	Fjjrgl.com	No	Hotels.com	No	
Aoyou.com	No	Lulutrip.com	No			

From Table 1, among the 8 online travel operators that provide the “Q & A” section, only five online travel operator platforms, Ctrip.com, Tuniu.com, Mafengwo.com, Qulxw.cn and, of which the “Q & A” section plays a role. However, Qulxw.cn and Lvmama.com do not provide any information of the questioner, so this paper takes the “Q & A” section of three well-known Chinese online travel operators, Ctrip.com, Mafengwo.com, Tuniu.com, as the source platform of the corpus.

Data crawling. Using Python language to edit the web crawler, with “outbound travel” as the key word, from the above four online travel operators “Q & A” section to crawl the “Q & A” data. The specific methods are as follows: first, collect the basic information of the page “Q & A” data through the Web page, set the collection field including title, questioner, question time and answer number, collect the above information to the local machine, establish a database and export the data; then, the answers of each question are collected, and the fields of collection are answer number, respondent ID, answer, answer time and like button number.

Data preprocessing. Firstly, the text is preprocessed to remove punctuation, obsolete words, convert fonts, mark part of speech and excess blank space to obtain the initial corpus.

#### 3.2.2. Feature selection

Since the part of speech in the corpus is very complex and there are some useless words such as prepositions and adverbs which may easily confuse the training results, we need to extract the part of speech from the processed corpus, that is, extract the feature words (Chang et al., 2018). Jieba library in Python is used to extract all the nouns in the separate words corpus to form a noun corpus. According to word frequency statistics of noun corpus, high-frequency words can reflect tourists’ risk perception of “outbound travel” to a certain extent.

In addition, keywords can reflect the main content or theme of the text, which means that keyword extraction is also an important part of feature selection, so TF-IDF method is used to extract keywords (Luo et al., 2016).

#### 3.2.3. Neural network language model

This research uses Word vector method to find tourism risk perception dimensions. To train word vectors, the required model parameters must be determined first. Hierarchical Softmax (Hierarchical Softmax) skip-gram model is adopted in the experiment. As for the choice of dimensions of word vector, generally speaking, too large dimension will lead to slow algorithm calculation speed and memory consumption, while too small dimension will cause a conflict of word vector mapping and affect training results, so the value of dimension between 100 and 200 is the best. For a corpus with less data, the smaller the dimension is, the more suitable it is. After word vector training, similar word vectors will cluster together and appropriate dimensions will make the clustering effect better. However, the model training effect cannot be seen from the general word vector text. In order to find the dimension suitable for the experiment, the model of dimension reduction should be adopted to judge. In the experiment, TSNE method is used to reduce the training models of different dimensions to 2-dimensional space, and the clustering effect of the trained models under different dimensions is observed. The dimension with the best clustering effect is selected as the dimension of the experimental training. Finally, the experimental parameters are as follows: the vector dimension is 200, the size of training window is 5, the skip-gram algorithm based on Hierarchical Softmax is adopted, the frequency of minimum word is 5, the number of iterations is 5 and the thread is 25.

#### 3.2.4. Word vector clustering and attribute classification

Clustering is an important part of data mining, and the purpose is to measure the similarity between data. There are many kinds of clustering algorithms, including K-means, DBSCAN, hierarchical clustering and so on. K-means algorithm is selected in the experiment, which is one of the most commonly used clustering algorithms. Its main characteristics are easy to understand, convenient to operate and fast operation speed.

Algorithm processes: randomly select k points as the center of mass of the data set; calculate the distance between each point in the data set and k centroid, and divide it into the cluster where the nearest centroid is; recalculate the sample mean of k clusters as the new center of mass; step 2 and step 3 are repeated until the center of mass no longer changes (Zhou et al., 2010).

#### 3.2.5. Results evaluation

After Word vector method is used to identify the dimensions of outbound tourism risk perception, the accuracy of the model is evaluated. The methodology as well as the procedures are presented in Table 2.

**Table 2:** Framework and process of data collection and analysis.

**Steps**	**Purpose**
Data crawling	Crawlers written in python are used to crawl online carrier “Q & A” data
Data preprocessing	Word segmentation(Font conversion+separate words +remove obsolete words+remove non-Chinese words)
Feature selection	Extracting nouns from split words
Word vectors training	Gensim module is used to train word vectors and TF-IDF method is used for clustering
Results evaluation	Evaluating the accuracy of the model and forming the final dimension of outbound travel risk perception

## 4. Results

### 4.1. Data crawling and preprocessing

#### 4.1.1. Data crawling

We use Python language to edit the crawler program, in January 2020, and take “outbound travel” as the key word, crawl the “Q & A” data from the “Q & A” section of Ctrip.com, Mafengwo.com, Tuniu.com, and Qyer.com. A total of 5, 292 questions and 27, 921 answers were collected. Each question corresponds to at least two or more answers. The maximum number of words in one question text is 556, and the maximum number of words in one answer text is 10,034. The minimum number of words per question text and per answer text is 1 word. Tourists’ descriptions of questions and answers vary greatly, without obvious rules, which is difficult for subsequent text processing. The average number of words per text for the questions and answers are 28.1 and 88.3 words. It is obvious that the description of the answers is more complex than the brevity of the questions.

#### 4.1.2. Data preprocessing

Python software is used to preprocess the crawled “Q & A” data, and the preprocessing process is shown in Table 3. First, the text is converted into sentences. Then, Jieba is used for word segmentation to remove stop words and non-Chinese words. Then remove all symbols in the sentence except Chinese. Finally, part of speech, such as name, verb, etc., is marked to form the initial corpus.

**Table 3:** Data prepossessing and feature extraction process.

**Pretreatment procedures**	**Text examples**
The original text	What is the travel strategy that Zhuhai goes to Macao’s exit port?
Word segmentation(Font conversion+separate words +remove obsolete words+remove non-Chinese words)	What is/Zhuhai/goes to/Macao/‘s/ exit /port/the travel strategy
Mark part of speech	What is/v zhuhai/ns goes to/v Macao/ns ‘s/ude1 exit/n port/n strategy/n
Feature extraction (nouns only)	zhuhai/ns Macao/ns exit/n port/n strategy/n
Separate words output	Zhuhai; Macao; exit; port; strategy

### 4.2. Feature selection

After separating the words, just keep nouns; use blanks to separate each noun, and output word segmentation results to form a noun corpus.

The word frequency of the noun corpus is counted, and the top 10 high-frequency words are shown in Table 4. By comparing the word frequency of questions and answers in Table 4, it is found that seven words are the same, such as exit, problem, entry, passport, Thailand, airport, and air ticket.

**Table 4:** Examples of high frequency words.

**Serial number**	**Question**		**Answer**	
	**Word**	**Statistics**	**Word**	**Statistics**
1	Exit	2,395	Author	5,191
2	Problem	531	Travel on a budget	4,726
3	Entry	393	Passport	3,174
4	Passport	346	Problem	3,126
5	Transaction	331	Entry	3,035
6	Hong Kong	328	Exit	2,969
7	Thailand	321	Air ticket	2,719
8	The Airport	281	Time	2,703
9	Transit	255	The airport	2,641
10	Air ticket	190	Thailand	2,394

TF-IDF method is used to extract keywords from the noun corpus, and the top 10 high-frequency keywords are shown in Table 5. As can be seen from the results of word frequency statistics and keyword extraction, the high-frequency words are almost the same as the keywords, with only a slight difference in order.

**Table 5:** Examples of Keywords.

**Serial number**	**Question**	**Weight**	**Answer**	**Weight**
1	Exit	1.220544882283646	Travel on a budget	0.26273114656069696
2	Entry	0.19627079869890054	Passport	0.14378798542052193
3	Passport	0.18538449679587063	Author	0.14172087876680942
4	Thailand	0.132834021340319	Entry	0.12815637002135838
5	Transit	0.12326014273584314	Exit	0.12793129520128044
6	Transaction	0.11967036041509846	air ticket	0.11598745852732927
7	Problem	0.11575325636923771	The airport	0.08657708859363139
8	The airport	0.10894882056863273	Thailand	0.08376195341174059
9	Hong Kong	0.10484952137925421	Transaction	0.07021630362569564
10	Air ticket	0.0958599201583654	Journey	0.06559656876093377

### 4.3. Word vector training

The vector dimension is 200, the size of the training window is 5, the skip-gram algorithm based on Hierarchical Softmax is adopted, the frequency of minimum word is 5, the number of iterations is 5, and the thread is 25. The word vector training is carried out to obtain the vector representation of all the words in the corpus and a word vector model. For example, the word vector representation of the word “exit” in the “question” section is −0.0046794955–0.14208734 0.12448783.

In addition, the cosine similarity is used to calculate the words that are similar to each keyword. The first five similar words of some keywords are shown in Table 6. It can be seen from Table 6 that the first five words similar to the keyword “exit” in the question are Shanghai, Shenzhen, San Francisco, Chicago and take a plane.

**Table 6:** Examples of word vector similar words.

**Question**	**Exit**	**Entry**	**Passport**	**Thailand**	**Transit**
1	Shanghai	London	The mainland	Landed	Bali
2	Shenzhen	Zhuhai	Shanghai	Phuket Island	Singapore
3	San Francisco	Urban district	Take a plane	Transit visa	Phuket Island
4	Chicago	Hong Kong airport	Situation	Hu Zhiming	China customs
5	Take a plane	Three countries	Mainland	Nepal	Aviation
**Answer**	**Travel on a budget**	**Passport**	**Author**	**Entry**	**Exit**
1	Mafengwo.com	Return ticket	Moda	Departure	Departure
2	Good ideas	Time effect	Tizhu	Visa	The title
3	Pennant	Notebook	Xieyao	Return ticket	Entry
4	Communication platform	Blank pages	Chujing	Deadline	Common sense
5	Block	Single	Fengfeng	Zhangzi	Visa

### 4.4. Clustering and attribute classification

**Figure 2:**
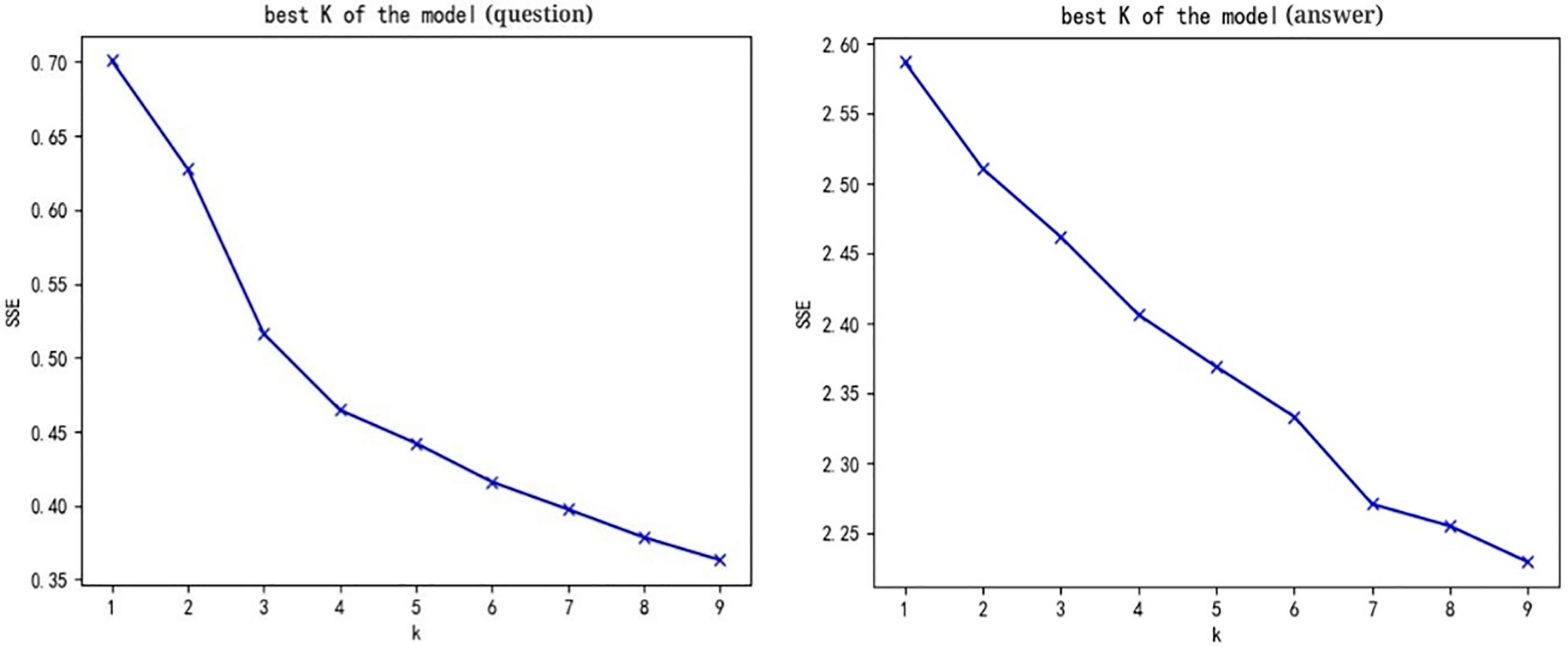
Results of the elbow rule.

According to the steps of cluster analysis of K-means algorithm, the elbow rule is adopted to determine the cluster number (K value). According to the elbow rule, the accuracy of each sample classification increases with the increase of K value. The clustering effect is closely related to the sum of squares of errors (SEE) value, which means, when the SSE is smaller, the aggregate effect of each cluster is better. As a result, the relationship between the value of k and the sum of the squares of the error shows a shape similar to that of a bent elbow. The best value is *K* at the elbow. The analysis results are shown in Figure 2. “Q & A” in the Word vector method, *k* = 7. When *k* = 7, *K*-means clustering is performed on the keywords in the training model using Python GenSim library, and 7 clusters were obtained. TF-IDF method is used to obtain the keywords of each cluster. See Table 7 for some keywords in question clustering and Table 7 for some keywords in answer clustering.

**Table 7:** Examples of keywords for the question cluster (Question, Top 10).

**Q**	**Type 1**	**Type 2**	**Type 3**	**Type 4**	**Type 5**	**Type 6**	**Type 7**
1	Phuket	The US visa	Guarantee	Identification of product	Telephone card	The French visa	Outbound tourism
2	Hong Kong airport	Service	Tuniu.com	Departure date	SLR cameras	Italian Visa	Plans
3	Car accidents	Join a group	Stores	Monoenergy	Join a group	Car rental	Novices
4	Connecting flight	Exit and entry	Service charge	Coupons	Huanggang	Barcelona	Langkawi
5	Duty-free shop	Round-trip ticket	Tour guide	You	Group tour	Zurich	Plans
6	Consign	Check-in	Solo	Charge membership fee	Join a group	Geneva	Tianyou
7	Transit visa	The boarding gate	Margin	Group fee	Bowery	Morocco	Dummy
8	Seal	The European tour	Adult	Coupons	Set up a team	The Czech republic	Travel on a budget
9	The return trip	Official website	Situation	Unionpay card	Groups	Vienna	single pass
10	Journey	Hotel	The order	Divorce certificates	Hotel	Portugal	Hero

From Table 7, the first category mainly revolved around airport related problems, corresponding to functional problems such as airport traffic and duty-free shopping. The second category is mainly service, mostly ticketing and visa issues. Both the first and second categories have traffic problems, and words related to flight precautions such as airport, consignment, and transfer account for a large proportion. It can be seen that because the plane is the main means of transportation out of the country, users account for a large proportion of questions about flights. The third category is financial, which is mostly related to expenses and includes guarantee fund, service fee, bank, and other keywords. The fourth category is similar to the third category. The key word is preferential treatment. Generally, outbound travel costs are high, and tourists are more sensitive to preferential information. The fifth category is the package tour, which is related to service. Since the risk of overseas tourism is much higher than that of domestic tourism and there are too many unknown factors, most tourists choose to travel in groups. There are many words related to group travel, such as leader, group and partner. The sixth category is tourism destination planning. Tourists have different preferences for the choice of tourist destinations. Tourists tend to choose Europe, Southeast Asia and other places as tourist destinations, and this category also includes destination visa issues. The seventh category has poor clustering effect, mainly including keywords related to trip and route planning, which belongs to planning risk.

From Table 8, the first category is related to planning and mostly answers to the choice of tourist destinations. The second category is related to the function, which is the answer to the questions about the tourist attractions and the project, including the introduction of various tourist projects and tourist attractions. The third category is similar to the fourth category, which is about traffic questions. The answers mainly focus on the matters needing attention in air travel, and the whole process of air travel is reflected in the answers. There are also a small number of functional responses. Tourist satisfaction can be improved by developing a detailed marketing strategy for tourist commodities. This category not only includes duty-free goods, security checks, and other airport duty-free shop commodity trading keywords, but also includes souvenirs, stores, and other overseas local souvenirs to buy. The fifth category is mainly about communication, finance, etc., which not only gives a detailed introduction to overseas communication, but also gives an introduction to overseas currency exchange and local currency circulation. At the same time, there are corresponding answers to questions about foreign currency and exchange rate conversion. There are also answers to service-oriented questions such as visas, and materials. The sixth category is mainly service problems. Tourists answered more questions about travel restrictions such as visas, passports, and information. The seventh category is communication problems, in which the customs and safety of tourist destinations are the issues that tourists pay more attention to. Tourists’ responses to such questions were comprehensive, and they also shared many travel experiences, such as route planning and hotel accommodations.

**Table 8:** Examples of keywords for the answer cluster (Answer, Top 10).

**A**	**Type 1**	**Type 2**	**Type 3**	**Type 4**	**Type 5**	**Type 6**	**Type 7**
1	New Swan Stone Castle	Thành phố Nha Trang	Book air tickets	Counter staff	Japanese Yen	The US visa	Join a group
2	Luzern	Duba	Meal ticket	Traveling case	Issuing bank	Transit passenger	Littering
3	Admit defeat for bet	The North of Thailand	Behind schedule	Flight information	Cashier’s desk	Leave the group	Senior
4	Reservation	Lanna	The taxi	Dutiable value	Travel network	Receipts	Local travel agency
5	Highway	Island tourism	Fu Jiapo	The Godmother	Network speed	Electronic ticket	Travel with friends
6	Through ticket	Wubu	Mini bus	Starbucks	Transportation card	*Via* advance	Singaporean
7	Night train	Erguna	Blagoveshchensk	Forbidden Object	A magnetic stripe card	Private passport	Occidental
8	Placed at the top	Famous attraction	Journey	Flight number	Phone card	Public security organs	Greyhound
9	Mount cook	Clefairy	Chiang rai	Declaration form	Power-consuming	Visa for study abroad	The questioner
10	White Russian	Tourists	Asiana	Departure hall	Cash	Entry visa	Halong bay

**Figure 3:**
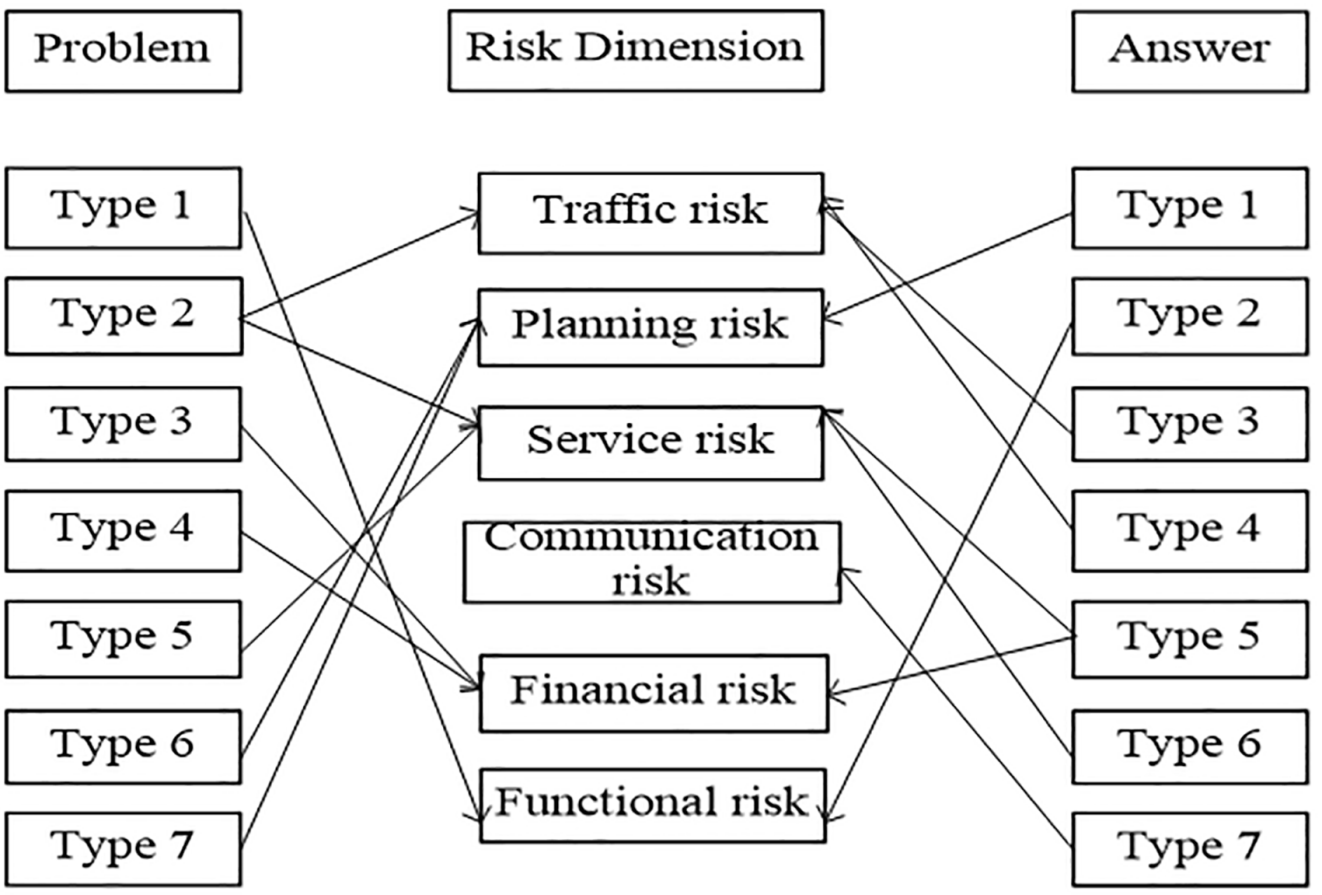
Attribute classification.

Based on the clustering results of questions and answers, Chinese tourists’ risk perception of outbound tourism can be mainly divided into traffic risk, planning risk, service risk, communication risk, financial risk, and functional risk. See Figure 3 for attribute classification.

It can be seen from the clustering results that the clustering of questions and answers does not reach the optimal effect, and there are problems such as category repetition and crossover.

### 4.5. Model accuracy evaluation

Evaluation of the model accuracy. The accuracy of the “Q & A” model before feature extraction are 59.18% (question) and 17.61% (answer) respectively. After feature extraction, the accuracy of the “Q & A” models are 70.69 and 76.69%, respectively. It shows that after the features are extracted, the accuracy of the model has been improved.

## 5. Discussion

### 5.1. Conclusion

The word vector method is used in this paper, with “outbound travel” as the key word, to crawl “Q & A” data from online travel operator platforms such as Ctrip.com and Tuniu.com, and then python is used to preprocess the data to form an initial corpus. Finally, the k-means method is used to cluster the data, and six outbound tourism risk perception dimensions are identified, which are traffic risk, planning risk, service risk, communication risk, financial risk and functional risk. It can be seen that there are some differences between our results and findings of previous researches. (1) Chinese tourists have the highest perception of traffic risk among the six outbound tourism risk perception dimensions discovered in the study, such as transportation issues such as boarding, transfers, and road safety. It is similar to the findings that flight delays and cancellations caused by airline operations are medium-high frequency risk events for Chinese tourists to travel abroad (Huang et al., 2022). (2) Chinese tourists pay more attention to service risks when travelling broad, such as scenic spots, hotels, and group tours in tourist destinations, and this result also applies to the study of Malaysian students’ risk perceptions when travelling to India (Khan et al., 2019), because low expectation about service quality develops a high perception of performance risk that negatively influences the purchase intention (Wood and Scheer, 1996; Garretson and Clow, 1999). (3) Financial risk refers to the possibility of not getting a corresponding return on the money spent in the process of travel, or the possibility of losing property (Roehl and Fesenmaier, 1992). Tourists are concerned about issues such as the protection of overseas property and consumption methods, as well as factors that influence tourist destinations’ itinerary. (4) Chinese tourists pay attention to their travel plan, that is, they make plans in advance when travelling abroad. Roselius (1971) argued that when a product does not perform according to expectations, people waste their precious time and convenience. (5) Chinese tourists are concerned about functional risk when traveling abroad, the objective factors affecting tourism safety perception include the social and natural environment in tourist destinations and the security situation of “food, housing, transportation, travel, shopping, entertainment” in the process of travel (Reisinger and Mavondo, 2005; Yüksel and Yüksel, 2007), and functional risk includes these aspects. (6) Chinese tourists have communication risk but the level of perception is not high, especially in group tours. On the one hand, due to the high quality of tour guides, the risk of communication is seldom paid attention to by tourists. On the other hand, experiencing exotic scenery is one of the motivations for Chinese tourists to travel abroad. Studies have proved that the culture of the destination country, the individual culture of the tourists, the culture of the destination country and the difference between the culture of the source country and the culture of the destination country are the cultural factors that affect the tourists’ behavior (Ng et al., 2007),

The classic outbound tourism risk perception dimensions are based on the integration of the research of Roehl and Fesenmaier (1992), Lepp and Gibson (2003), Lepp et al. (2011), including 9 dimensions of hardware facilities, money and finance, physical health, psychological reasoning, expectation satisfaction, social relations, time cost, political events, and cultural differences and obstacles. Comparing our research results with the 9 dimensions, it is found that Chinese tourists do not consider the risks such as physical health, time cost, and political events.

First, Chinese outbound tourists have insufficient perception of physical health risks. The possible reason for this phenomenon is that the main destinations of Chinese tourists’ outbound travel are Thailand, Japan, the United States, the Maldives, Canada, the Netherlands, Australia and other countries. In the perception of Chinese tourists, these countries are generally safe. However, even one major incident can malign the image of a destination (Yang et al., 2017). Chinese visitors have no perception of physical risk does not mean that these nations and areas should not emphasis on providing a better environment in terms of safety and security. Therefore, providing physical safety and security is a must to sustain the growth of tourism of a destination. Second, the time cost is not within the risk perception of Chinese outbound tourists. This situation is likely to be that many Chinese outbound tourists are people who are retired or have not entered the workplace. This group of people has a lot of free time and generally does not consider the cost of time. According to the *2019 China Cross-Border Travel Consumption Report* jointly released by Ctrip.com and Mastercard, the age distribution of Chinese outbound tourists is mainly located in the 50s and 60s (retirees) and 90s and 00s (most of them did not enter the workplace) (Luo, 2019). Third, Chinese outbound tourists do not attain a good understanding of the risks of political events. The reason for this is that tourists actively avoid countries and regions with high risk of political incidents, such as Mali, Libya, Syria, Afghanistan, etc., when choosing a destination.

### 5.2. Implications

Chinese tourists lack awareness of risks such as physical health, time costs, and political events. In view of risk perception of Chinese outbound tourists, relevant Chinese government agencies and enterprises ought to take the following measures:

1. Visitors should steer clear of any situations where political hazards put their safety at risk. Thus, the extreme danger, high risk, medium risk, low risk, and minor risk may be marked on the international tourism risk map as they develop. Remind Chinese travelers to select safe countries and regions before they decide where to go and apply for visas. Before going, try to reduce the likelihood of security problems, which is mostly brought on by political threats.
2. It is critical to caution tourists to pay attention to physical risk prevention. When choosing an outbound travel destination, Chinese outbound tourists generally prefer nations and areas with low levels of travel risk, which leads them to pay less attention to preventing health risk when travelling. To ensure travel safety, picking low-risk nations and areas is by no means sufficient. For instance, two Chinese tourists perished from hypothermia at a picturesque Icelandic location in January 2020. As a result, it is important to constantly remind Chinese tourists traveling abroad to pay attention to the prevention of physical health hazards and increase personal safety protection.
3. Relevant Departments ought to remind visitors to step up risk-prevention efforts related to property. This study shows that Chinese outbound tourists have given careful consideration to property risks, but they might also experience heinous crimes like robbery and property losses abroad. This demonstrates the necessity for Chinese tourists travelling abroad to raise their level of property risk avoidance awareness. Online travel companies, travel agents, and other affiliated enterprises, as well as government organizations like the Chinese Ministry of Culture and Tourism, must public reports on accidents involving outbound travel and make the risks associated with such travel more widely known.
4. Tourists need to be more alert to the dangers of traffic. According to this study, Chinese tourists who go overseas are particularly worried about traffic dangers, including those related to flights, transfers, and road safety, yet they nonetheless frequently experience traffic accidents when they are away from home. For example, on September 15, 2019, an SUV driven by a Chinese tourist collided with a bus carrying 34 passengers on the Indian Ocean Highway in Western Australia. The accident caused two deaths and one serious injury to the three Chinese tourists on the SUV. Remind tourists of traffic risks. If tourists choose to travel by car, they should ensure that they are familiar with local traffic rules.

### 5.3. Limitations and future research directions

1. Due to the limitation of anti-reptile technology, the corpus of training word vectors based on “Q & A” data is not large. In the future, we will consider directly using the online comments of tourists to delete and select the risk-perceived comments from the comments to generate larger-scale data.
2. Feature selection in the experiment only extracts nouns, and does not perform different analyses for words of different parts of speech. In the future, in addition to the analysis of nouns, verbs, adjectives, etc., can also be analyzed separately, and the analysis results of different parts of speech corpora can be compared.
3. The data of this study crawled in January 2020, which portrays the risk perception of Chinese tourists’ outbound travel before the new crown epidemic. After the outbreak of the new crown epidemic, the National Health Commission of the People’s Republic of China has listed overseas as high-risk areas, and Chinese tourists cannot travel abroad in principle.

## Data availability statement

The original contributions presented in the study are included in the article/supplementary material, further inquiries can be directed to the corresponding author.

## Author contributions

CY prepared the first draft. YZ prepared materials and collected data. All authors contributed to manuscript revision and read and approved the submitted version.

## Funding

This project was financed by the National Social Science Fund of China (Project no.: 20BGL161).

## Conflict of interest

The authors declare that the research was conducted in the absence of any commercial or financial relationships that could be construed as a potential conflict of interest.

## Publisher’s note

All claims expressed in this article are solely those of the authors and do not necessarily represent those of their affiliated organizations, or those of the publisher, the editors and the reviewers. Any product that may be evaluated in this article, or claim that may be made by its manufacturer, is not guaranteed or endorsed by the publisher.
